# Identification of potential target genes of breast cancer in response to Chidamide treatment

**DOI:** 10.3389/fmolb.2022.999582

**Published:** 2022-11-08

**Authors:** Han Han, Xue Feng, Yarui Guo, Meijia Cheng, Zhengguo Cui, Shanchun Guo, Weiqiang Zhou

**Affiliations:** ^1^ Department of Pathogen Biology, Shenyang Medical College, Shenyang, China; ^2^ Department of Biomedical Statistics, Graduate School of Medicine, Osaka University, Suita, Japan; ^3^ Department of Environmental Health, University of Fukui School of Medical Science, Fukui, Japan; ^4^ RCMI Cancer Research Center, Xavier University of Louisiana, New Orleans, LA, United States

**Keywords:** Chidamide, breast cancer, RNA-seq, bioinformatics, TP53, ACLY, PPARG

## Abstract

Chidamide, a new chemically structured HDACi-like drug, has been shown to inhibit breast cancer, but its specific mechanism has not been fully elucidated. In this paper, we selected ER-positive breast cancer MCF-7 cells and used RNA-seq technique to analyze the gene expression differences of Chidamide-treated breast cancer cells to identify the drug targets of Chidamide’s anti-breast cancer effect and to lay the foundation for the development of new drugs for breast cancer treatment. The results showed that the MCF-7 CHID group expressed 320 up-regulated genes and 222 down-regulated genes compared to the control group; Gene Ontology functional enrichment analysis showed that most genes were enriched to biological processes. Subsequently, 10 hub genes for Chidamide treatment of breast cancer were identified based on high scores using CytoHubba, a plug-in for Cytoscape: TP53, JUN, CAD, ACLY, IL-6, peroxisome proliferator-activated receptor gamma, THBS1, CXCL8, IMPDH2, and YARS. Finally, a combination of the Gene Expression Profiling Interactive Analysis database and Kaplan Meier mapper to compare the expression and survival analysis of these 10 hub genes, TP53, ACLY, PPARG, and JUN were found to be potential candidate genes significantly associated with Chidamide for breast cancer treatment. Among them, TP53 may be a potential target gene for Chidamide to overcome multi-drug resistance in breast cancer. Therefore, we identified four genes central to the treatment of breast cancer with Chidamide by bioinformatics analysis, and clarified that TP53 may be a potential target gene for Chidamide to overcome multi-drug resistance in breast cancer. This study lays a solid experimental and theoretical foundation for the treatment of breast cancer at the molecular level with Chidamide and for the combination of Chidamide.

## 1 Introduction

Breast cancer is a major malignant tumor that endangers women’s health. According to statistics, there are about 2.81 million confirmed cases of breast cancer worldwide in 2021, accounting for 30% of all female cancer cases, and the mortality rate reaches 15% among female malignant tumors (Siegel et al., 2021). Nearly 85% of breast cancer patients are estrogen receptor (ER) positive. ER is a prototypical member of the nuclear receptor superfamily, which plays a central role in cell proliferation, survival and invasion; it is also a transcription factor that affects the expression of target genes through genomic and non-genomic pathways, a process that is critical for cell growth and proliferation and tumor cell growth, proliferation and survival (Heldring et al., 2007; Kojetin et al., 2008; Hanker et al., 2020). In recent years, anti-hormonal therapy targeting estrogen receptors has improved the treatment of breast cancer to some extent. However, tumor resistance by common clinical therapeutic agents has greatly reduced the therapeutic efficacy (Elder et al., 2006; Huang et al., 2017). Therefore, the search for effective targets for targeted drug action has been a challenge for researchers (Waks and Winer, 2019; Garcia-Martinez et al., 2021; Mehraj et al., 2021).

Chidamide, a novel structural, isoform-selective histone deacetylase inhibitor that is a promising anticancer agent, and our previous experiments have also demonstrated that Chidamide is a new generation HDACi with lower toxicity and higher efficacy than other HDACi such as SAHA (Han et al., 2017; Zhou et al., 2021), and that it is more effective in combination therapy, especially in difficult-to-treat advanced breast cancer, and may help overcome its drug resistance (Munster et al., 2011; Kern, 2016; Yeruva et al., 2018). However, the research and application of the molecular mechanisms of their antitumor pharmacological effects are still in their infancy and cannot meet all the criteria required for targeted drugs. Therefore, more and more in-depth studies on the antitumor effects of Chidamide are needed to find more accurate targets of action and explore more and better utilization of its value.

Traditional single studies are hampered by the limited number of samples and cannot systematically analyze key genes and their molecular functions in complex biological processes. While bioinformatics analysis based on high-throughput platforms is a powerful tool, whole transcriptome sequencing, also known as RNA-seq technology, is the sequencing of all RNAs reverse transcribed into cDNA libraries in cells using second-generation high-throughput sequencing technology, which can effectively obtain the entire transcript information of an organism in a specific physiological context (Prokop et al., 2018; Van Dijk et al., 2018; Ulintz et al., 2019).

The application of RNA-seq can discover drug targets and mechanisms of action and facilitate the progress of drug development (Wang et al., 2016; Zhang et al., 2017). Therefore, we selected ER-positive breast cancer MCF-7 cells and used RNA-seq to analyze the gene expression differences of breast cancer cells treated with Chidamide, and performed functional enrichment of potential genes to clarify the drug targets of Chidamide for the treatment of breast cancer, laying the foundation for the treatment of malignant tumors, especially breast cancer, and the development of new drugs.

## 2 Materials and methods

### 2.1 Materials

The human breast cancer cell line MCF-7 was purchased from the American Cell Conservatory (ATCC); all cell culture reagents, RPMI-1640 medium, fetal bovine serum, penicillin and streptomycin were purchased from Thermo, United States ; CHID was purchased from Sigma.

### 2.2 Cell culture

All tests were set up in triplicate wells, and three replicates of each experiment were guaranteed. The human breast cancer cell line MCF-7 was cultured in RPMI-1640 medium containing 15% fetal bovine serum, 100 U/mL penicillin, and 100 μg/ml streptomycin. Breast cancer cells at logarithmic growth stage were collected in 15 ml centrifuge tubes, centrifuged at 1,000 rpm for 5 min, resuspended in medium containing 15% fetal bovine serum, and the cell suspension was adjusted to 5.0 × 10^5^ cells inoculated in 6-well plates. The final concentration of Chidamide was adjusted to 20 μmol/l, and the cells were collected after receiving Chidamide for 24 h in a control group with an equal volume of cell culture medium.

### 2.3 Establishment of transcriptome libraries

Total RNA was isolated from cells using Trizol (invitrogen) and RNA purity was assessed using the ND-1000 Nanodrop. Each RNA sample had an A260:A280 ratio above 1.8 and an A260:A230 ratio above 2.0. RNA integrity was assessed using an Agilent 2,200 Tape Station (Agilent Technologies, United States ) and each sample had a RIN above 7.0.

Subsequently, the purified RNA was fragmented to approximately 200 bp according to the instructions of the NEBNext® Ultra™ RNA Library Prep Kit for Illumina (NEB, United States ).), the purified RNA was subjected to first- and second-strand cDNA synthesis, followed by splice ligation and low-circulation enrichment.

### 2.4 Transcriptome data analysis

A large amount of sample double-end sequencing data was obtained through the Illumina platform. Given the impact of data error rate on the results, Fast QC was used to check the quality of the initial RNA-Seq data, Trimmomatic software was used to pre-process the raw data for quality, to remove joints and low quality reads, and to summarize the statistics of the number of reads throughout the quality control process. The Clean Reads were sequenced against the specified reference genome using hisat2 (http://daehwankimlab.github.io/hisat2/download/) to obtain information on the position of the reference genome or gene, as well as information on sequence characteristics specific to the sequenced samples.

### 2.5 Differential expression analysis

The statistically significant DE genes were obtained by an adjusted *p*-value threshold of <0.05 and |log2 (fold change)| > 1 using the DEGseq. Finally, a hierarchical clustering analysis was performed using the R language package gplots according to the TPM values of differential genes in different groups. And colors represent different clustering information, such as the similar expression pattern in the same group, including and colors represent different clustering information, such as the similar expression pattern in the same group, including similar functions or participation in the same biological process.

### 2.6 Gene ontology terms and KEGG pathway enrichment analysis

All differentially expressed mRNAs were selected for GO and KEGG pathway analyses. GO was performed with KOBAS3.0 software. GO provides label classification of gene function and gene product attributes (http://www.geneontology.org). GO analysis covers three domains: cellular component (CC), molecular function (MF) and biological process (BP). The differentially expressed mRNAs and the enrichment of different pathways were mapped using the KEGG pathways with KOBAS3.0 software (http://www. genome.jp/kegg).Visualize the results using the R language package gplots.

### 2.7 Protein interaction network and hub gene identification

The protein interaction network of DEGs was analyzed using the STRING (https://string-db.org/) online database; then the PPI network was visualized using Cytoscape software (version 3.6.0) and key targets (hub genes) were calculated from the network using CytoHubba, a plug-in for Cytoscape (Chin et al., 2014). There are 11 topological analysis methods in CytoHubba, and we chose the Degree Method to select the top 10 genes in terms of the number of nodes (nodes in a protein interaction network represent proteins, edges represent interactions between proteins, and the number of edges connecting a node to other nodes represents the importance of that node in the protein interaction network) as potential key genes for regulation.

### 2.8 Validation of central genes in clinical samples

To confirm the expression of hub genes in clinical samples, we screened 10 hub genes for expression in the online platform Gene Expression Profiling Interactive Analysis (GEPIA), a web server for analyzing RNA-sequencing expression data of 9,736 tumors and 8,587 normal samples from the TCGA and GTEx projects, using standard processing pipelines. *p*-values < 0.01 and fold changes >1 were considered thresholds for expression between tumor and normal samples. In addition, survival curves for the survival of hub genes between high and low expression patients were validated in the Kaplan Meier plotter (http://kmplot.com/analysis/). The Kaplan Meier plotter is a meta-analysis-based tool for the discovery and validation of survival biomarkers. It was able to assess the impact of 54 k genes on survival in 21 cancer types. The largest datasets include breast cancer (n = 6,234) and lung cancer (*n* = 3,452) (Nagy et al., 2018).

### 2.9 Single nucleotide variants and pathway activity of validated genes

UALCAN (http://ualcan.path.uab.edu/) is an online platform for cancer data analysis and mining mainly for The Cancer Genome Atlas (TCGA) database. UALCAN provides easy and fast access to the publicly available TCGA canceromics data, as well as the identification of tumor biomarker molecules or online simulation validation of target gene expression in tumors and prognostic survival analysis.

The Genomic Cancer Analysis (GSCA) database is a web-based platform for genomic cancer analysis (http://bioinfo.life.hust.edu.cn/web/GSCALite/). The platform integrates cancer genomics data from TCGA for 33 cancer types as well as normal tissue data from GTEx. Hub genes validated by GEPIA and Kaplan Meier mapper were further validated for expression in breast cancer and corresponding normal tissues using the UACLAN and GSCA online databases, and further analyzed by GSCA for genetic alterations and methylation (Tang et al., 2017).

### 2.10 Statistical analysis

All statistical analyses were performed in R (v3.5.2), and *p* < 0.05 was considered statistically significant. Fisher’s exact test and likelihood ratio test were employed for comparison of variables.

## 3 Results

### 3.1 Raw sequencing data quality and transcriptome sequencing statistics

**FIGURE 1:**
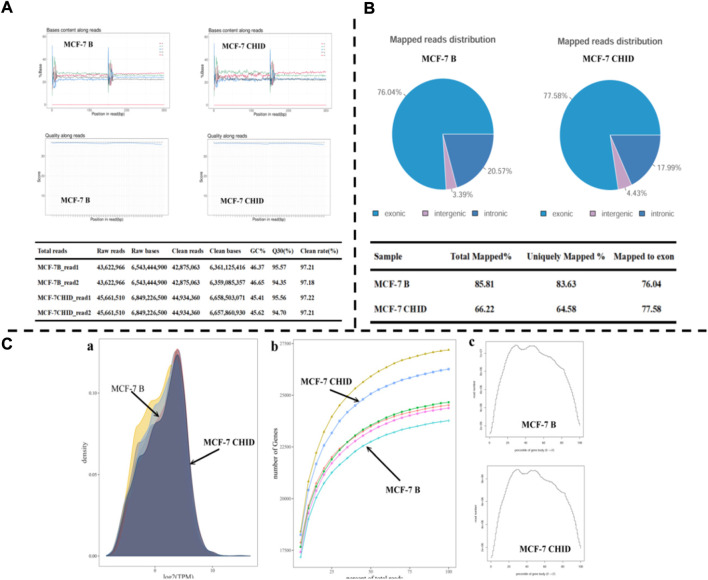
Transcriptome analysis of MCF-7 after Chidamide treatment. **(A)**: Quality inspection chart of initial data. **(B)**: Mapping information of RNA-Seq; **(C)**: a: Gene expression density map; b: Sequencing saturation results for each sample. The horizontal coordinate indicates the amount of sequencing data (expressed as a percentage) and the vertical coordinate indicates the number of genes detected. c: Transcript coverage homogeneity. The horizontal coordinates of the graph indicate the gene length (expressed as a percentage) and the vertical coordinates indicate the number of reads in the region).

The results showed that the base quality of most of the sequences was above 30, indicating good sequencing quality. The four lines of sequencing samples were parallel and close to each other, and no base shift was observed. The percentage of GC was more than 45% (Figure 1A).

The total number of reads in MCF-7 Basal group and MCF-7 CHID group were obtained as 43,622,966 and 45,661,510, respectively; the percentage of clean reads obtained by filtering the lower quality data was 97.2% and 97.2%, respectively (Figure 1A).

The sequencing data were compared with the reference genome using HISAT2 software to make a comprehensive evaluation of the coverage area and depth of coverage of the sequencing data. The results showed that more than 66% of the sequences were matched to the reference genome, 64% of the sequences were matched to only one position, and more than 76% of the reads were matched to exonic regions (Figure 1B).

### 3.2 Analysis of differentially expressed genes

The TPM density distribution pattern was used to examine the gene expression pattern of the samples as a whole; the TPM density distribution pattern showed that moderately expressed genes accounted for the majority, and low and high expressed genes accounted for a small proportion. Sequencing saturation analysis showed that the relative error decreased as the sampling proportion increased, and the regions of sequencing results were saturated; the distribution of sequencing Reads on gene coverage showed an overall distribution trend of lower sequencing coverage at the 5′ end and 3′ end, and higher in the middle, with credible sequencing results (Figure 1C).

**FIGURE 2:**
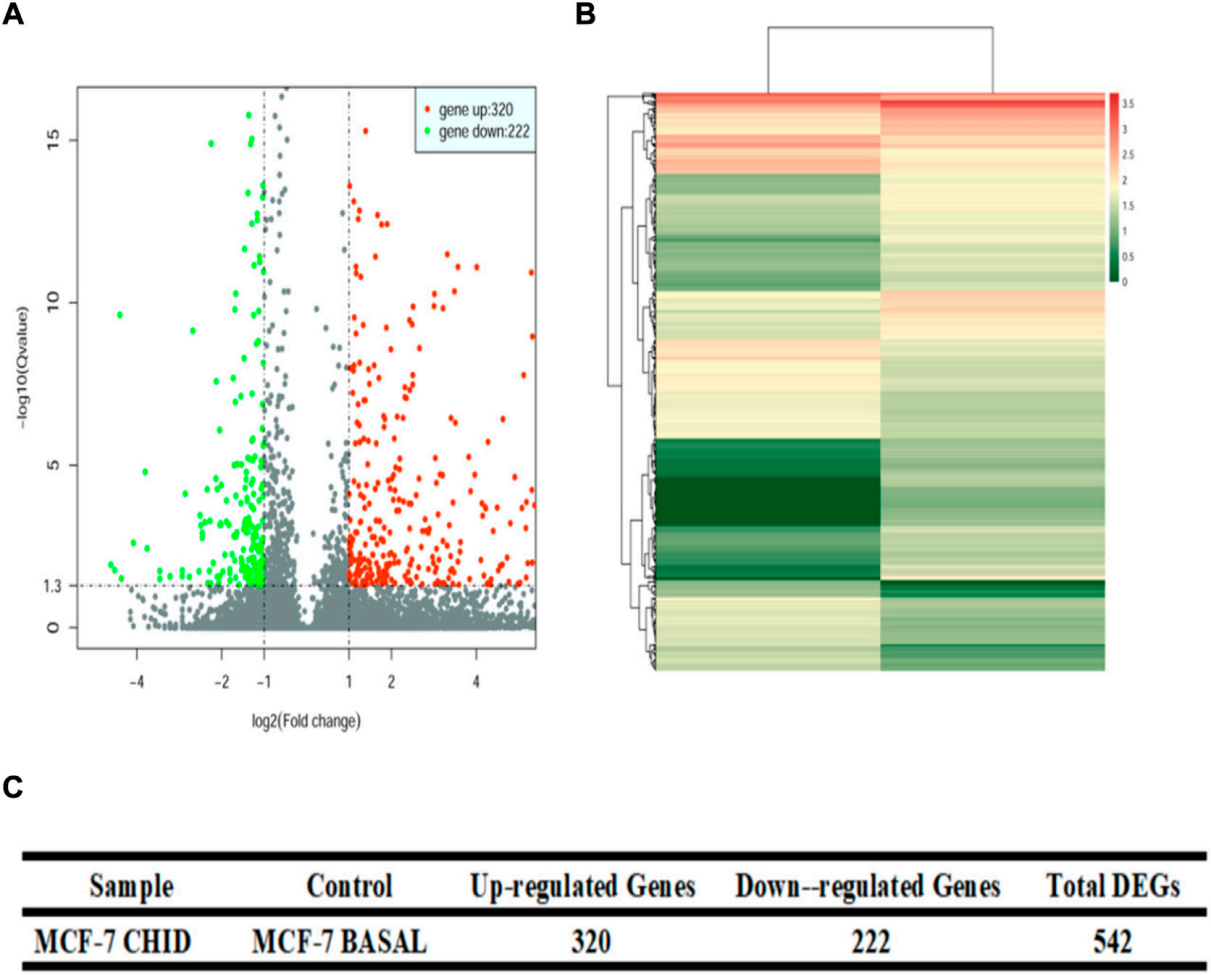
Volcano plot and Heat Map of differential expression gene. **(A)**: Volcano plot for differential gene expression analysis between samples. The horizontal coordinates represent the fold change of gene expression in different samples; the vertical coordinates represent the statistical significance of the difference in gene expression, red dots indicate significantly upregulated genes and green indicates significantly downregulated genes. **(B)**: Heat map of differential gene expression analysis between samples. Colors represent log 10 (expression value + 1) values. **(C)**: Quantitative analysis of differential gene expression between samples.

Differential analysis of gene expression was performed by DESeq (version 1.18.0). The statistical results of differentially expressed genes showed that, compared with the control group, the statistical results of differentially expressed genes showed that the MCF-7 CHID group expressed 320 upregulated genes and 222 downregulated genes (Figure 2). The volcano and heat maps were also able to show that the MCF-7 CHID group expressed more upregulated genes than downregulated genes in the control group.

### 3.3 Differential gene ontology energy set analysis

**FIGURE 3:**
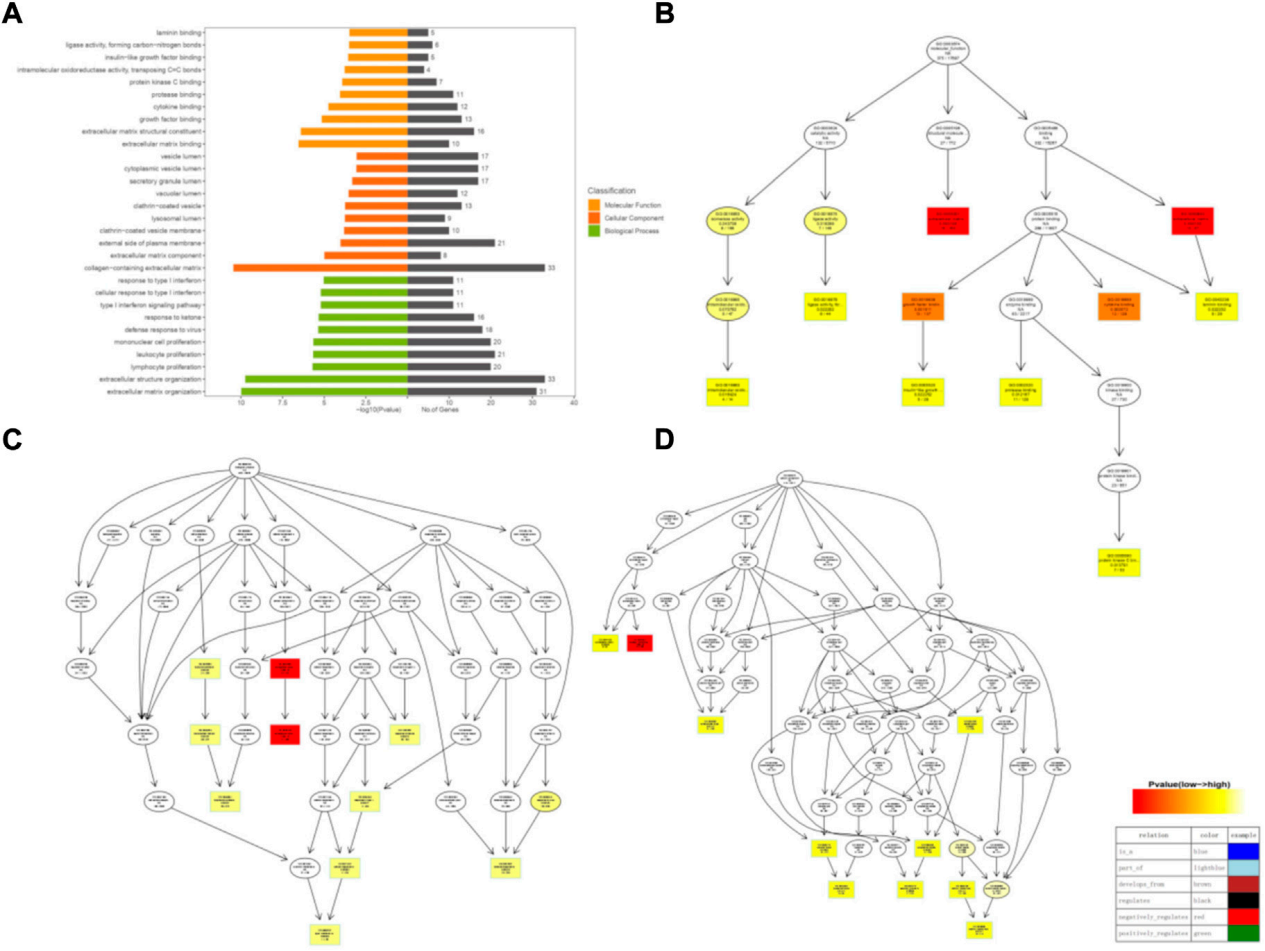
GO enrichment analysis of DEGs. **(A)**: Differential Gene GO Enrichment Distribution Map. Differential Gene GO Enrichment Directed Acyclic Graph. The darker the color, the higher the enrichment level. **(B)** GO molecular function analysis; **(C)** GO biological process analysis; **(D)** GO cellular component analysis.

The results of GO enrichment analysis revealed that in the MCF-7 CHID group (Figure 3), a total of 685 GO entries were enriched in GO molecular function (MF), and in the Top10, and the enriched molecular functions mainly included binding activity (laminin binding, insulin-like growth factor binding, protein kinase C binding, protease binding, cytokine binding, growth factor binding, extracellular matrix binding), enzymatic activities (ligase activity, intramolecular oxidoreductase activity), and structural components of the extracellular matrix, suggesting that CHID may be involved in supporting and protecting MCF-7 cell tissues as well as in cell growth and adhesion; 443 GO Term were enriched in the GO cellular component (CC), and among the Top 10, the cellular components with relatively high number of enriched genes mainly included vesicle-containing lumen; cytoplasmic vesicle lumen; secretory granule lumen; vacuolar lumen; clathrin−coated vesicle; lysosomal lumen; clathrin−coated vesicle membrane; followed by external side of plasma membrane; extracellular matrix components; and collagen-containing extracellular matrix, indicating that CHID affects gene expression in a variety of cellular vesicle lumen and extracellular matrix in MCF-7 cells. A total of 4231 GO Terms were enriched in the GO Biological process (BP), and among the Top10, they mainly included immune regulation (response to type I interferon; cellular response to type I interferon; type I interferon signaling pathway) response to ketones; defense response to viruses; immune cell proliferation (mononuclear cell proliferation; leukocyte proliferation; lymphocyte proliferation); extracellular structural organization; extracellular matrix organization and other biological processes, suggesting that CHID-treated MCF-7 cells may affect a variety of immune-modulatory response processes.

### 3.4 KEGG pathway enrichment analysis

The metabolic and signaling pathways involved in differential genes were analyzed using KEGG enrichment, where the vertical coordinate is the KEGG Pathway entry, the horizontal coordinate is the Rich factor, the size of the dot in the graph indicates the number of differential genes annotated to the pathway, and the color indicates the pathway’s significant *p*-value.

**FIGURE 4:**
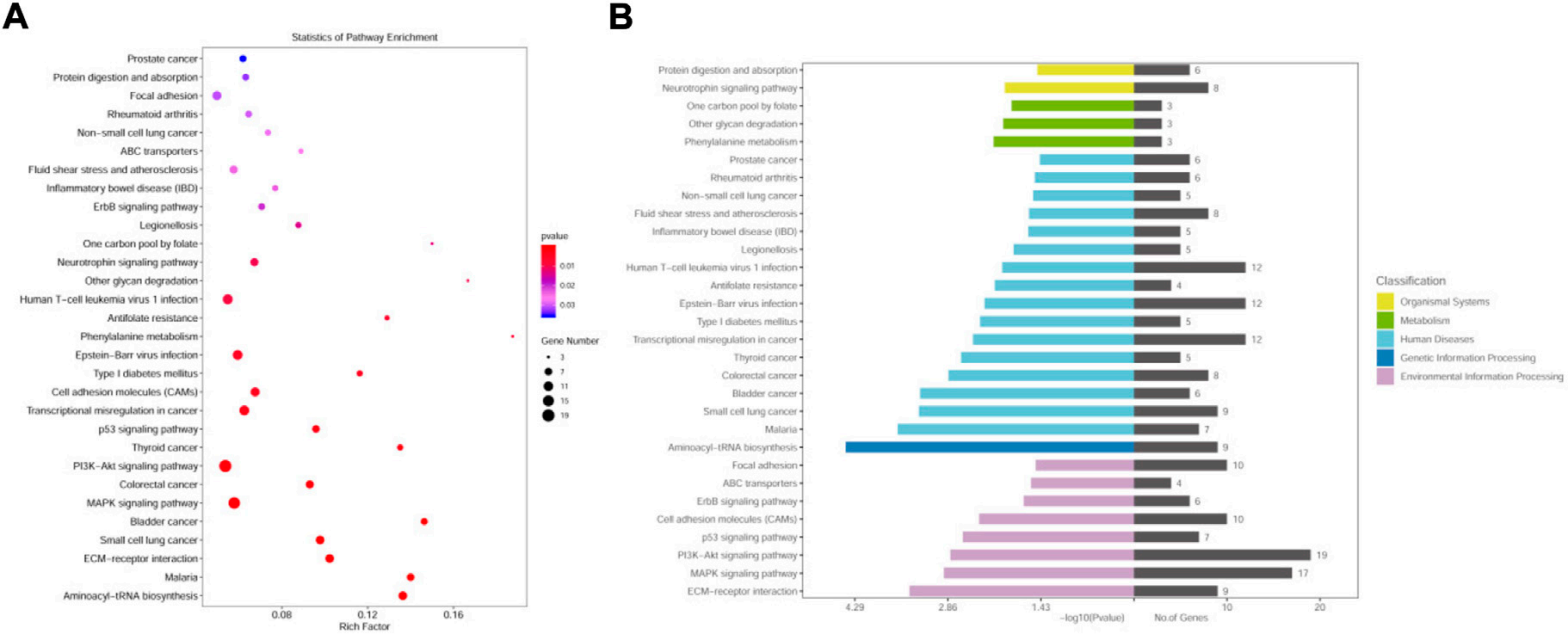
KEGG pathway enrichment analysis of DEGs. **(A)**: Differentially expressed gene KEGG pathway bubble map. The horizontal coordinates indicate the proportion of enriched differential genes to the background genes of the pathway, and the vertical coordinates indicate the name of the pathway; the size of the dots in the graph indicates the number of enriched differential genes, and the color indicates the *p*-value. The size of the dots in the graph indicates the number of enriched differential genes, and the color indicates the *p*-value. **(B)**: Distribution of differentially expressed gene KEGG pathway enrichment.

The MCF-7 CHID group was enriched in 265 signaling pathways, with the top 30 signaling pathways being enriched (Figure 4). Among the enriched Top 30 signaling pathways, 50% were related to cytology of the human diseases, 30% to environmental signaling, and the remainder to metabolic and biological systems, with these signaling pathways being primarily engaged in human diseases. (e.g., Malaria, Small cell lung cancer, Bladder cancer, Colorectal cancer, Thyroid cancer, Transcriptional misregulation in cancer, Type I diabetes mellitus, Epstein-Barr viral infections, Anti-folate resistance, Human T−cell leukemia virus 1 infection, Legionellosis, Inflammatory bowel disease (IBD), Fluid shear stress and atherosclerosis, Non-small cell lung cancer, Rheumatoid arthritis, Prostate cancer, etc.), environmental signaling-related pathways (e.g., Focal adhesion, ABC transporters, ErbB signaling pathway, cell adhesion molecules (CAMs), p53 signaling pathway, PI3K-Akt signaling pathway, MAPK signaling pathway, ECM-receptor interaction, etc.), metabolism (e.g., Phenylalanine metabolism, Other glycan degradation, One carbon pool by folate, etc.), biological systems (e.g., Neurotrophin signaling pathway, Protein digestion and absorption, etc.), genetic signaling processes (Aminoacyl−tRNA biosynthesis), etc. This suggests that MCF-7 CHID is engaged in a wide range of cellular biologic activities. Several signaling pathways in Top30 are closely related to cell proliferation, implying that CHID may participate in MCF-7 cell regulatory processes *via* cell proliferation-related signaling pathways.

### 3.5 Protein interaction network and functional annotation analysis

**FIGURE 5:**
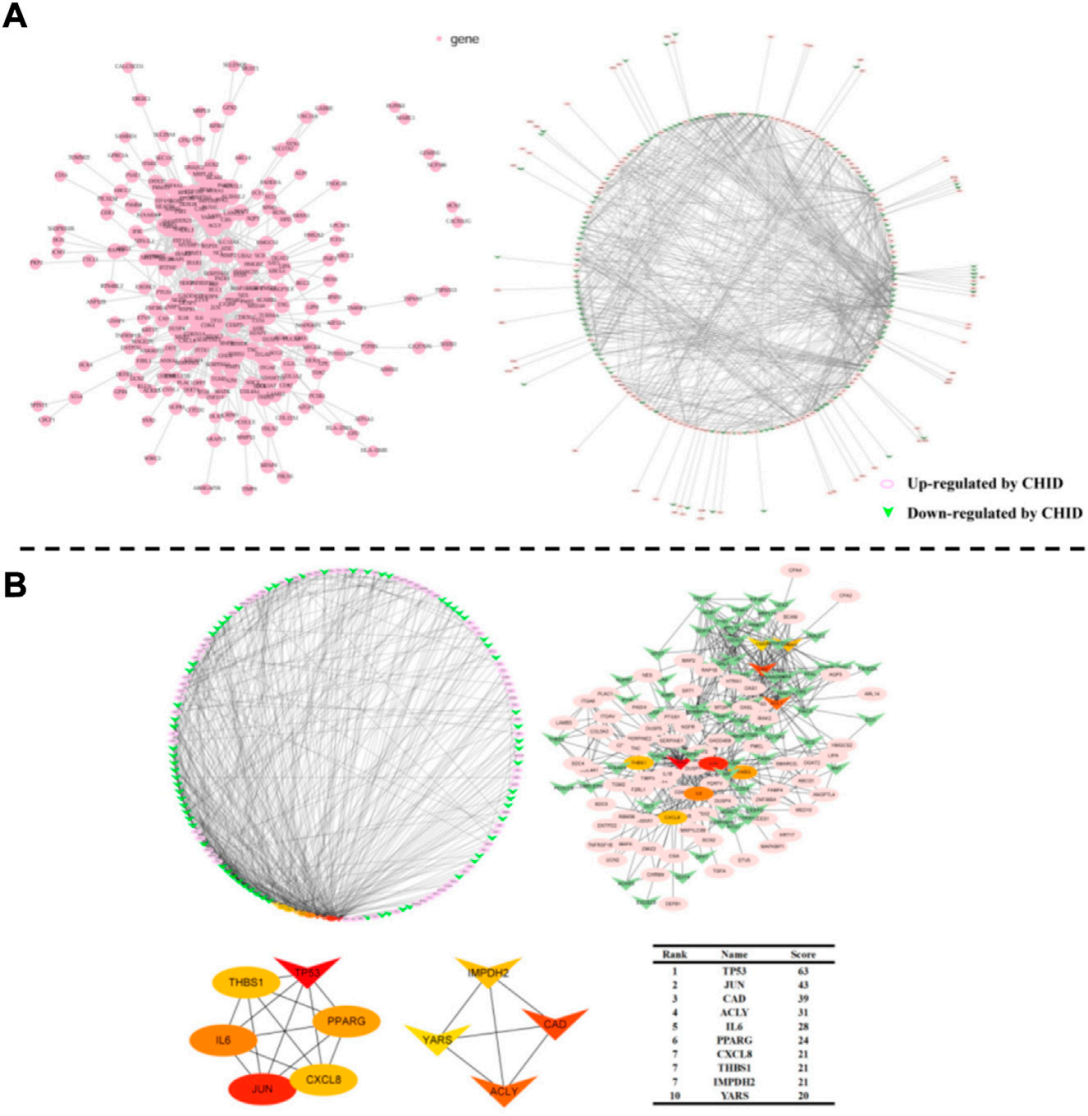
**(A)**: Protein-Protein Interaction Network Analysis Chart. PPI network exported from STRING and visualized in Cytoscape. A node represents a gene. The genes increased in MCF-7 by Chidamide were shown in pink color; at the same time, the genes that down-regulated in MCF-7 by Chidamide were posted in triangles. **(B)**: Selection of hub genes. PPI network was analyzed by CytoHubba, a plugin of Cytoscape. A node represents a gene. The hub genes with higher scores were demonstrated with the larger sizes.

To understand the interaction of DEGs at the protein level, protein-protein interaction (PPI) networks were constructed by Multiple Protein in the online database STRING. 542 proteins encoded by DEGs were able to form a complex protein interaction network (Figure 5A). Cytoscape was used to visualize and analyze the PPI network (Figure 5A). CytoHubba is a plug-in for Cytoscape to calculate the hub genes in the network.TP53, JUN, CAD, ACLY, IL-6, PPARG, CXCL8, THBS1, IMPDH2, and YARS were identified as hub genes of the network based on high scores (Figure 5B).

### 3.6 Validation of key genes in clinical samples

**FIGURE 6:**
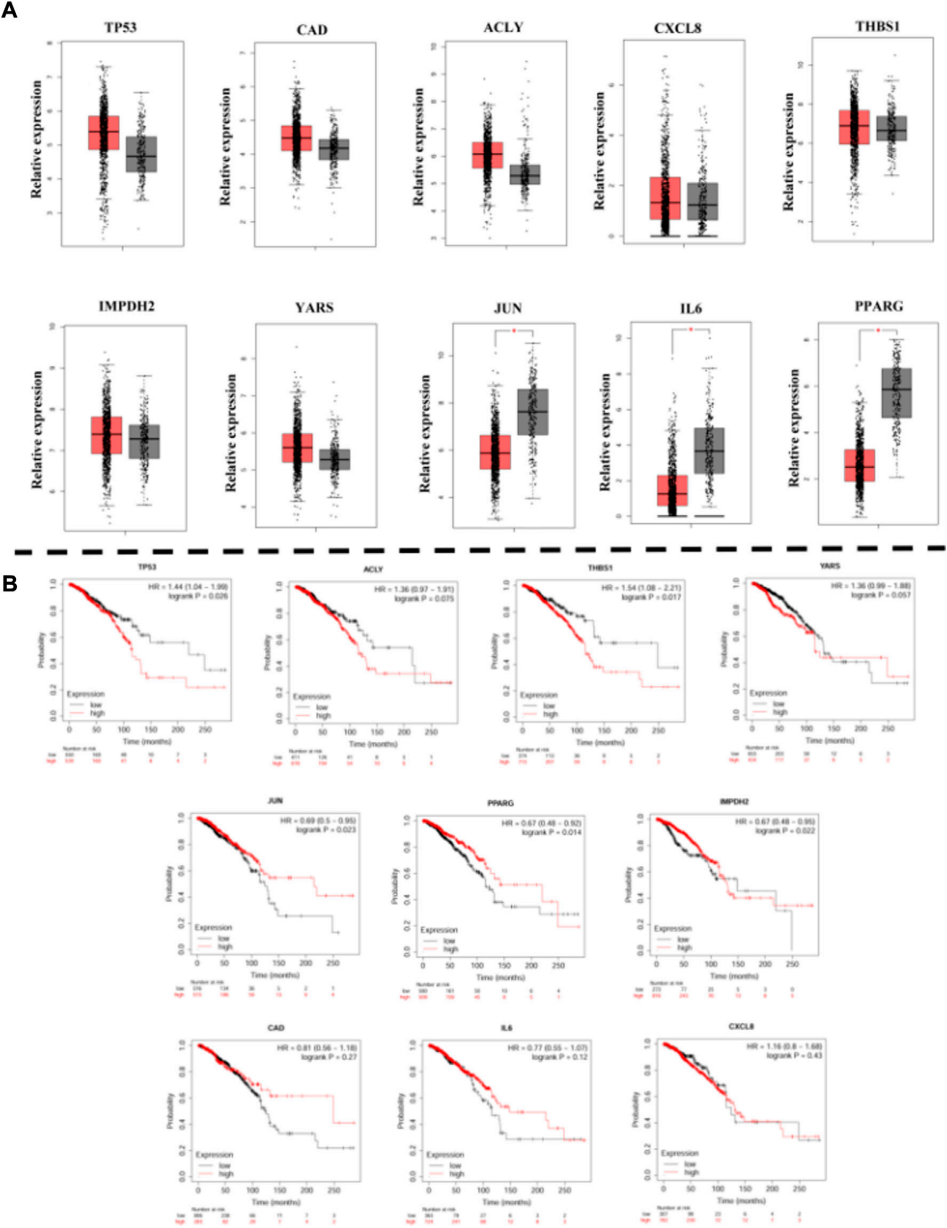
**(A)**: The expression of hub genes in BREAST samples of TCGA. **(B)**: Kaplan-Meier curves displaying OS of BREAST.

To verify the aberrant expression of the identified DEG in breast cancer cells, we determined the expression of pivotal genes in clinical samples from GEPIA (Figure 6A). The mRNAs of TP53, CAD, ACLY, IMPDH2, and YARS were found to be highly expressed in tumor cells among these genes and down-regulated by Chidamide treatment; the mRNAs of THBS1 and CXCL8 were highly expressed in tumor cells, and the expression was higher after treatment with Chidamide. The mRNAs of JUN, IL-6 and PPARG were reduced in tumor cells and up-regulated by Chidamide treatment. Therefore, we hypothesized that the regulation of TP53, CAD, ACRY, IMPDH2, YARS, JUN, IL-6, and PPARG by Chidamide is beneficial to the treatment of breast cancer.

To determine the impact of DEGs on the prognosis of breast cancer patients, the Kaplan-Meier mapper was used to predict the prognostic value of 10 hub genes. Our study found that high expression of TP53, ACLY, THBS1 and YARS was associated with worse overall survival (OS) in breast cancer patients (Figure 6B); while low expression of JUN, PPARG and IMPDH2 was associated with worse OS in breast cancer patients; while expression of CAD, IL6 and CXCL8 was not associated with OS in breast cancer patients (CAD, *p* = 0.27. IL6, *p* = 0.12; CXCL8, *p* = 0.43).

### 3.7 Single nucleotide variants and pathway activity of validated genes

Combining the results validated in clinical samples with those obtained from previous differential gene screens (Supplementary Figure S1), we considered TP53, ACLY, JUN, and PPARG as potential candidate genes. Subsequently, analysis of the expression of these genes in breast cancer and corresponding normal tissues using the UACLAN and GSCA online databases revealed that the results were consistent with those obtained by GEPIA and Kaplan Meier mapper (Supplementary Figure S2, Supplementary Figure S3), further suggesting that TP53, ACLY, JUN, and PPARG ACLY, JUN, and PPARG genes have the potential to become therapeutic candidates. Subsequently, the GSCA online database was used to analyze the differential expression of these genes at different stages of breast cancer development (Supplementary Figure S4).

**FIGURE 7:**
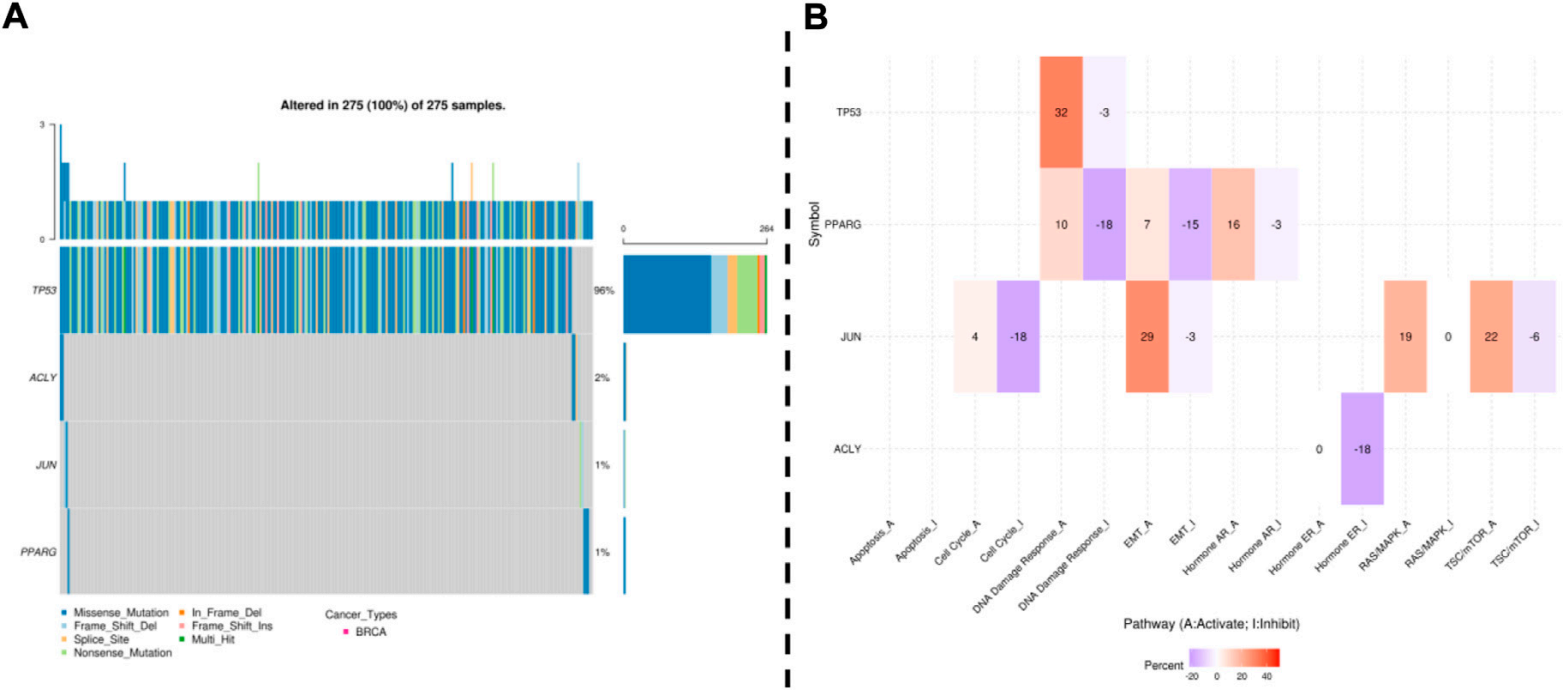
GSCA online database was selected to analyze the single nucleotide variation and pathway activity of validated genes. **(A)**: Waterfall plots, give a single nucleotide variation in BREAST gene sets. **(B)**: Effects of the validated gene on cell pathway activity, gene expression was divided into 2 groups (High and Low) by median expression, the difference of pathway activity score (PAS) between groups was defined by student *t*-test, *p*-value was adjusted by FDR, FDR ≤0.05 was considered as significant. The pathway activity module presents the correlation of gene expression with pathway activity groups (activation and inhibition) that are defined by pathway scores.

Next, the genetic alterations and methylation of the above candidate genes were further analyzed by GSCA. Single nucleotide variants (SNVs) were detected in 4 hub genes in 275 samples. The frequency of SNVs in TP53 was the highest among the 4 hub genes, reaching 96% in 275 samples. Missense mutations were the most important type of mutation (Figure 7A). In addition, we considered the effect of hub genes on pathway activity. The results showed that Hub genes are involved in the regulation of DNA damage response, apoptosis, EMT and hormone signaling pathways, which are important signaling pathways for tumorigenesis and development (Figure 7B).

## 4 Discussion

The effective investigation of the pharmacological mechanism of action using contemporary sequencing technology has become one of the dominant development paths of current research, with the penetration of high-throughput sequencing technology into numerous fields (Sumner et al., 2007; Wang et al., 2009; Shi et al., 2010). Drug screening has entered the high-throughput age, and an increasing number of novel targets and prospective targets have been found, largely attributable to the advancement of genetic databases and database analysis by automated bioinformatics operating systems (Kim et al., 2013; He et al., 2015; Ujihira et al., 2015; Zheng et al., 2016). In this study, for the first time, we analyzed the gene expression of histone deacetylase inhibitors in breast cancer cells and controls using RNA-seq, and found that the difference in gene expression of Chidamide was more significant in breast cancer cells compared to SAHA, which is consistent with our previous experimental results (Han et al., 2017; Zhou et al., 2021). Since nearly 85% of breast cancer patients were ER positive and recent results showed that ER transcription factors are strongly associated with breast cancer metastasis and invasion, we finally selected Chidamide for subsequent analysis of MCF-7 cells.

Differential analysis of gene expression by DESeq showed that the MCF-7 CHID group expressed 320 upregulated genes and 222 downregulated genes as compared to the control group. The volcano and heat maps also revealed that the MCF-7 CHID group had more up-regulated genes than the control group. According to the findings, CHID influences the biological activity of breast cancer cells through controlling the expression of these differential genes.The analysis of the biological process (BP), cellular component (CC), and molecular function (MF) of up-regulated and down-regulated DEGs suggested that CHID may perform multiple functions by regulating different DEGs, thus participating in the regulation of biological processes such as apoptosis, adhesion and immune regulation.

Among these genes, TP53, JUN, CAD, ACLY, IL-6, PPARG, THBS1, CXCL8, IMPDH2, and YARS were identified as key differential genes for Chidamide treatment of breast cancer based on high scores. To verify whether the identified differential genes are aberrantly expressed in breast cancer cells and the impact on the prognosis of breast cancer patients, we examined the expression of pivotal genes in clinical samples from GEPIA and performed survival analysis of the above-mentioned pivotal genes, as well as for different stages of breast cancer disease progression, and identified TP53, ACLY, PPARG and JUN as potential candidate genes significantly associated with Chidamide treatment of breast cancer. Subsequent analysis of the expression of these genes in breast cancer and corresponding normal tissues using the UACLAN and GSCA online databases was found to be consistent with the results obtained above, further suggesting the potential of TP53, ACLY, JUN, and PPARG genes to be candidate therapeutic genes.

Abnormal tumor cell growth and proliferation require a large number of biomolecules that build cellular components, of which fatty acid anabolism has a particularly important role (Wakil and Abu-Elheiga, 2009; Currie et al., 2013). Increased fatty acid synthesis has been shown to be strongly associated with poor prognosis in a variety of tumors (Corbet et al., 2016; Li and Zhang, 2016). While normal tissues and cells rely primarily on exogenous lipid intake to meet their needs, tumor cells prefer to use acetyl coenzyme A for *ab initio* fatty acid synthesis (Santos and Schulze, 2012; Cheng et al., 2018). In many tumors such as lung, prostate, bladder, breast, and colon cancers, ACLY is pathologically overexpressed or has enhanced enzymatic activity (Khwairakpam et al., 2015; Granchi, 2018; Icard et al., 2020). In addition, its catalytic production of acetyl CoA is also a donor of acetyl groups, and acetylation modification of histones plays an important role in the regulation of gene expression, DNA replication and DNA damage repair (Wellen et al., 2009; Chen et al., 2019; Orsó and Burkhardt, 2020). Therefore, inhibition of ACLY activity or interference with ACLY can effectively inhibit the *ab initio* synthesis of lipids and histone acetylation, thereby inhibiting tumor cell growth, making ACLY targeting for tumor inhibition a potential research hotspot (Feng et al., 2019; Chen et al., 2020). Our study showed that Chidamide could inhibit the highly expressed ACLY mRNA in breast cancer cells, therefore, it is speculated that ACLY could be a potential drug target for Chidamide in the treatment of breast cancer.

Another drug target we have identified for the treatment of breast cancer is peroxisome proliferator-activated receptor gamma (PPARG), a ligand-dependent transcription factor that is a member of the nuclear hormone receptor superfamily and plays an important role in regulating glucose and lipid metabolism *in vivo*. The activation of PPARG has been increasingly shown to inhibit the proliferation, migration and invasion of breast cancer cells, while its down-regulation promotes the progression of cachexia in breast cancer patients (Janani and Ranjitha-Kumari, 2015; Yang et al., 2020; Kandel et al., 2021). This is consistent with our results in clinical samples compared in the GEPIA database, where mRNA for PPARG was lowly expressed in breast cancer cells compared to normal individuals. Kaplan-Meier mapper prediction studies also confirmed that low expression of PPARG was associated with worse OS in breast cancer patients. In contrast, the expression of PPARG was significantly increased in breast cancer cells treated with Chidamide. In addition, it has been shown that PPARG agonists also enhance the activity of Histone deacetylase Inhibitors (HDACi), which may act as epigenetic regulators to exert anti-cancer effects (Mishra et al., 2014; Aouali et al., 2015). Therefore, we speculate that the histone deacetylase inhibitor Chidamide could positively feedback PPARG to act as an inhibitor of breast cancer cells.

The most interesting of these genes is the TP53 gene. TP53 is the gene most closely related to human tumors discovered so far, with two isoforms: wild type (Wtp53) and mutant type (Mtp53). Wild type TP53 plays an important role in maintaining normal cell growth and inhibiting malignant proliferation, while in contrast, mutant TP53 gene affects normal cell division and promotes abnormal proliferation of tumor cells, eventually leading to carcinogenesis, and human malignant tumors are most commonly of the mutant type (Bourdon, 2007; Hong et al., 2014; Stein et al., 2019). We compared TP53 gene expression in clinical samples using the GEPIA database and found that TP53 mRNA was highly expressed in breast cancer cells compared to normal individuals. Kaplan-Meier mapper prediction study found that high TP53 expression was associated with worse OS in breast cancer patients. When analyzed in the GSCA database, the SNV frequency of TP53 was found to be the highest among the four hub genes, reaching 96% in 275 breast cancer samples, with missense mutations being the most important type of mutation. The positive expression of the mutant TP53 gene is closely related to the recurrence and prognosis of breast cancer, and its overexpression suggests strong proliferative activity, poor differentiation, high malignancy, invasive ability and high metastasis of lymph nodes in tumor cells. Most scholars believe that in many different types of tumor cells, activation of wild-type P53 can make them sensitive to some chemotherapeutic drugs; while mutant P53 can specifically activate MDR-1/P-gp initiation and can increase the expression of MDR-1, MRP genes and make tumor cells produce multidrug-resistant (MDR) (Breier et al., 2013; Chen et al., 2014; Zhang et al., 2020). The high expression rate of mutant TP53 gene suggests that breast cancer cells are insensitive to third-generation aromatase inhibitors (Zhao et al., 2017; Bellazzo et al., 2018; Zhou et al., 2019). Therefore, targeting therapy against TP53 has become one of the main directions of cancer treatment research at present. It has been shown that Chidamide inhibits the transcription and translation of mutant TP53 (Li et al., 2019; Zhang et al., 2022). In progressive diffuse large B-cell lymphoma (DLBCL) patients with Rituximab-resistant TP53 mutations, Chidamide may play a chemo-sensitizing role by inhibiting the transcription and translation of mutant TP53 and up-regulating the surface expression of CD20 antigen in lymphoma cells (Li et al., 2019). Our results also confirm the ability of Chidamide to inhibit the expression of mutant TP53 mRNA in breast cancer cells. It is worth mentioning that some other studies have shown that Chidamide is able to cause the onset of cell cycle arrest by stimulating TP53 expression, which eventually triggers apoptosis (Liu et al., 2016; Yuan et al., 2019; Cao et al., 2021). This result, which is highly consistent with our previous experiments, showed that Chidamide-induced breast cancer cell death is closely related to apoptosis and transitional cell autophagy, and TP53 plays a central role in this process. And the related experimental results are also reflected in the experimental results of other HDACI such as SAHA that we have studied (Feng et al., 2017; Zhou et al., 2021). Another report showed that Chidamide in combination with Doxorubicin (DOX) induced p53-mediated cell cycle arrest and apoptosis and inhibited MDR in breast cancer cells (Cao et al., 2021). The combination of Decitabine and Chidamide not only had a powerful beneficial effect on acute myeloid leukemia (AML) symptoms but also restored TP53 mutations in AML patients (Zhang et al., 2022). Does this suggest that Chidamide can inhibit the gene activity of mutated TP53 and restore the gene activity of wild-type P53 thereby overcoming breast cancer MDR. TP53 may be a potential target gene for Chidamide to overcome breast cancer MDR.

Several HDACIs, including Chidamide, are currently in clinical trials, both as monotherapy and in combination with other agents. As single agents, they show promise in the treatment of hematologic malignancies, but they are not as effective in solid tumors due to acquired drug resistance and impact on the target site. Identifying the target of a single drug is a prerequisite for designing a multidrug combination approach. The restoration of mutant TP53 wild-type function in breast cancer cells by Chidamide identified in our study provides a theoretical basis for the combination of Chidamide.

In conclusion, Chidamide, as a chemically new HDACi-like drug with a novel structure, has been shown to inhibit breast cancer, but its specific mechanism has not been fully elucidated. In this study, we identified four hub genes to the treatment of breast cancer with Chidamide through bioinformatics analysis, and clarified that TP53 may be a potential target gene for Chidamide to overcome MDR in breast cancer. This study lays a solid experimental and theoretical foundation for the treatment of breast cancer with Chidamide at the molecular level and for the combination of Chidamide.

## Data Availability

The datasets presented in this study can be found in online repositories. The names of the repository/repositories and accession number(s) can be found in the article/Supplementary Material.

## Supplementary material

SUPPLEMENTARY FIGURE S1 The expression of differential gene results statistics.

SUPPLEMENTARY FIGURE S2 UALCAN online database was selected to analyze the expression of hub genes in BREAST samples of TCGA.

SUPPLEMENTARY FIGURE S3 GSCA online database was selected to analyze the expression of hub genes in BREAST samples.

SUPPLEMENTARY FIGURE S4 GSCA online database was selected to analyze the expression of tendency in pathologic stages.
