# Supplementary figures and images for: Identification of potential target genes of breast cancer in response to Chidamide treatment
Source: Front Mol Biosci. 2022 Nov 8;9:999582. doi: 10.3389/fmolb.2022.999582 (PMC9679413; doi:10.3389/fmolb.2022.999582)

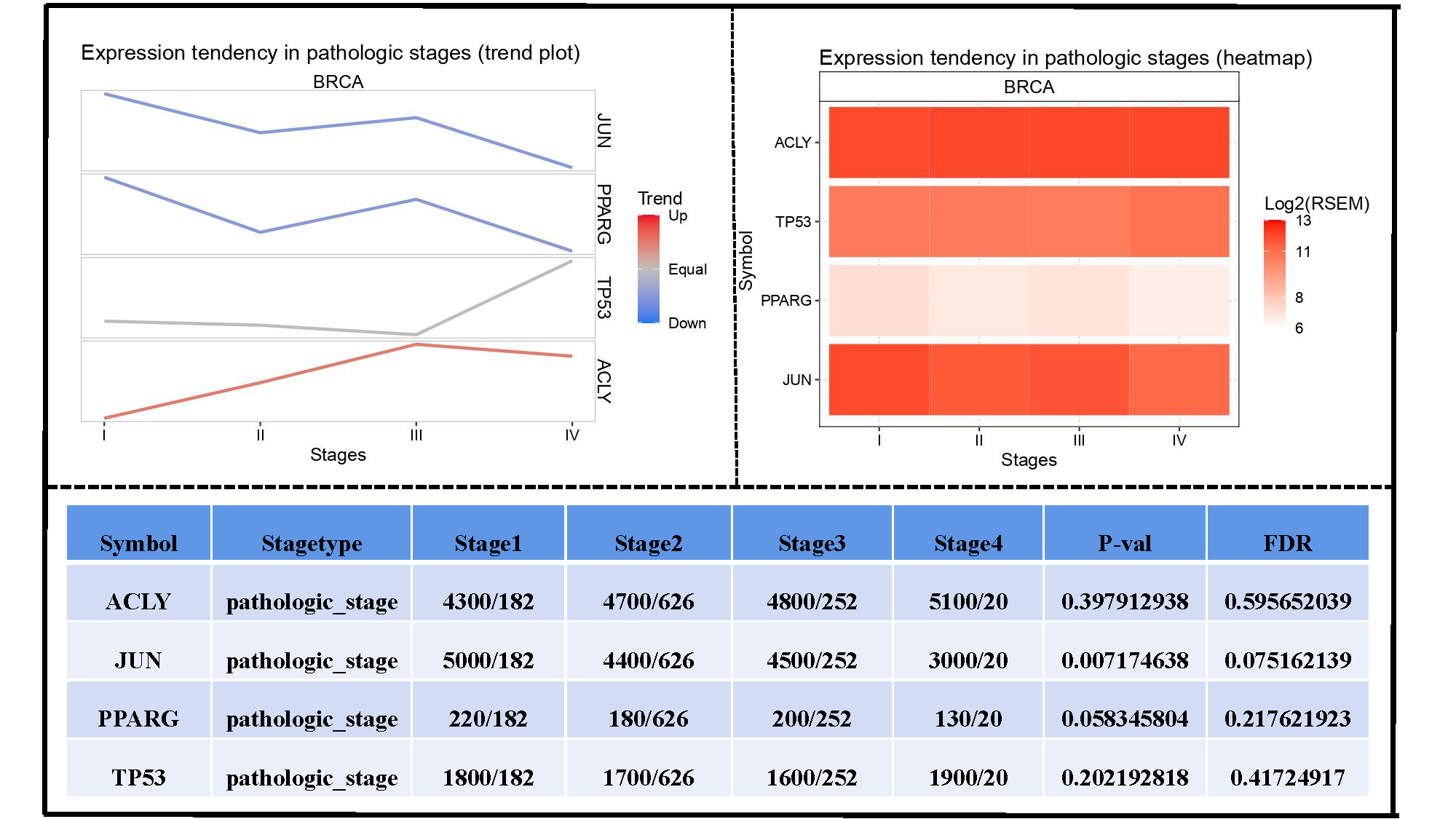

Supplement: Supplementary file 2 [file Image4.TIF]

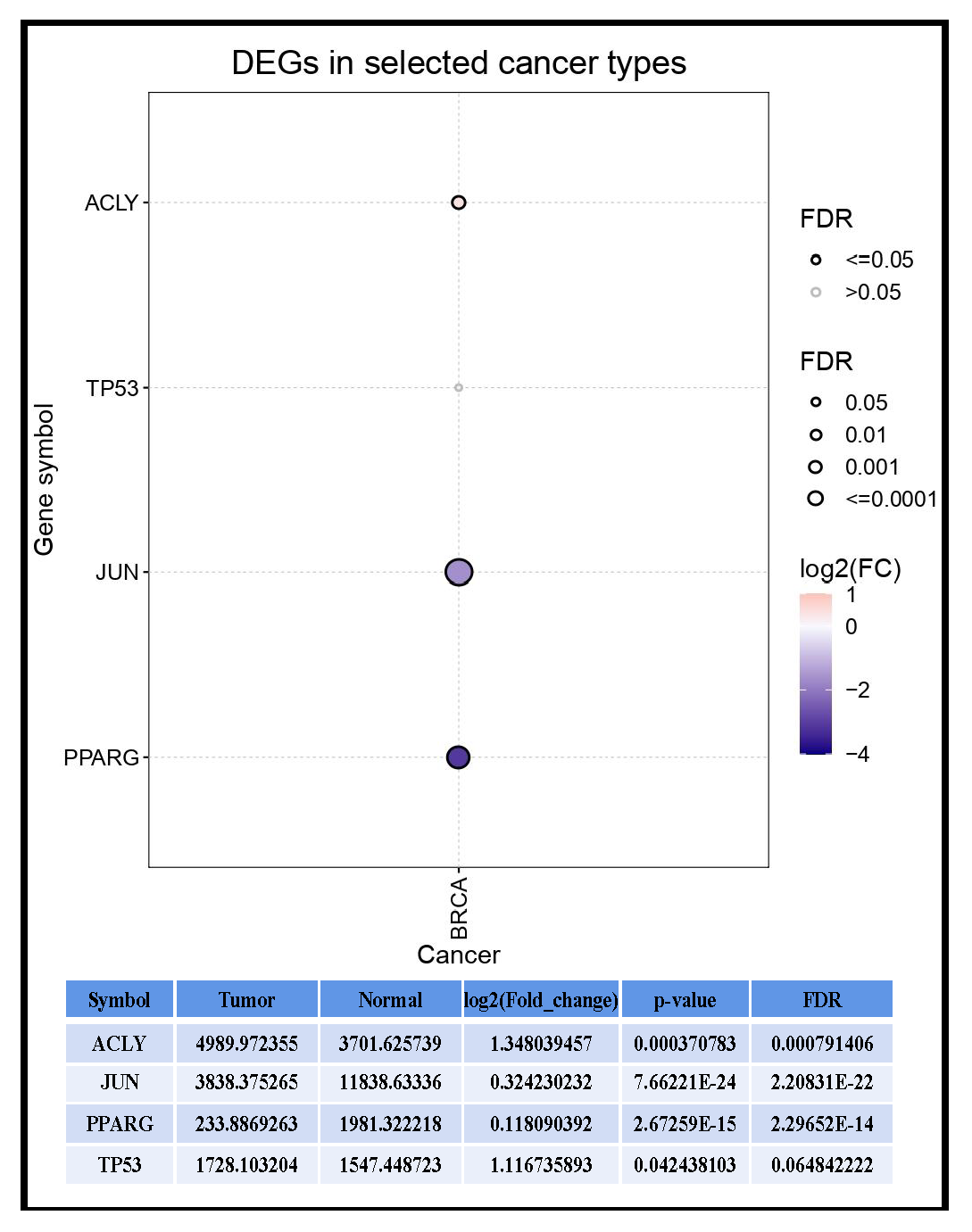

Supplement: Supplementary file 3 [file Image2.TIF]

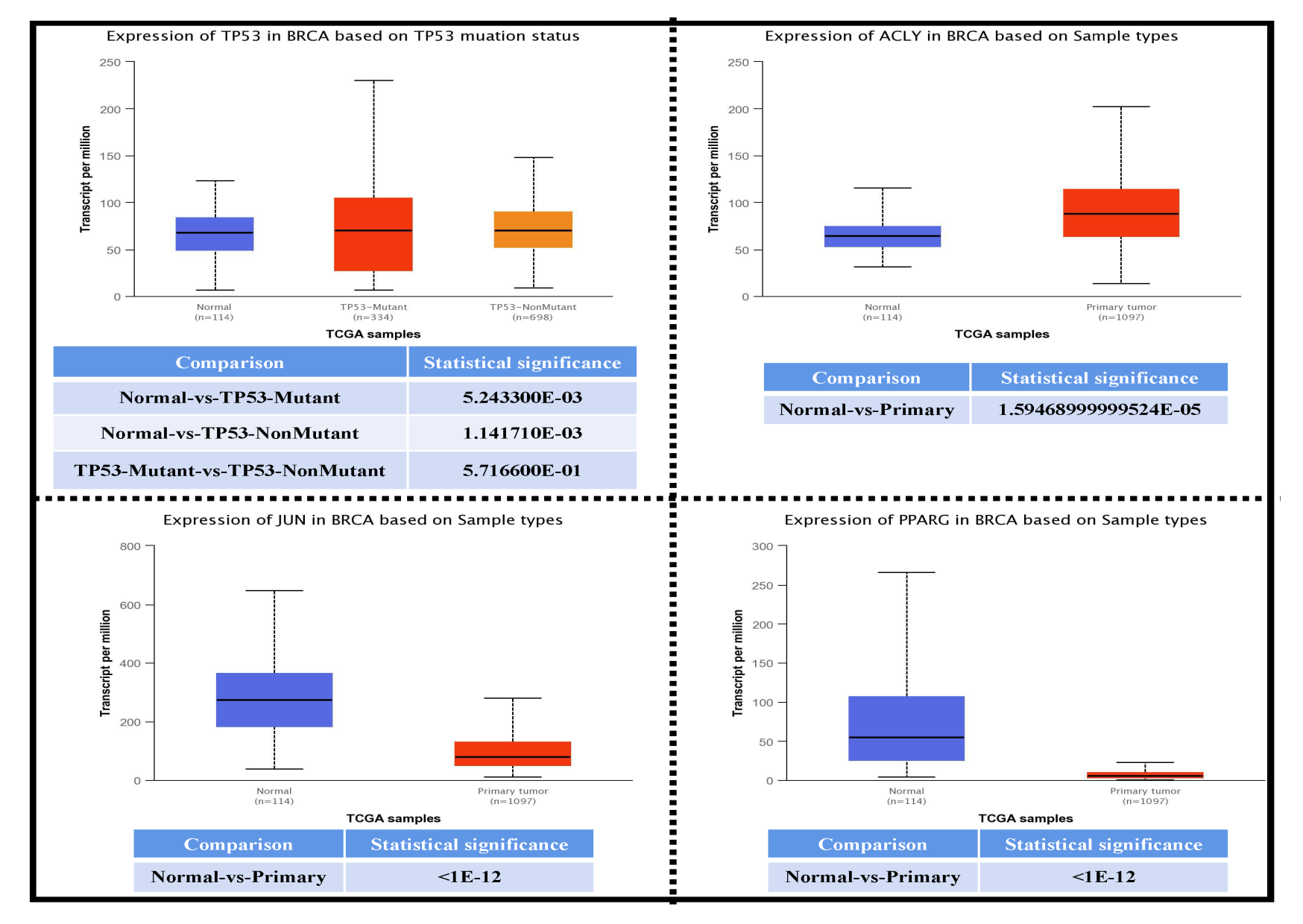

Supplement: Supplementary file 4 [file Image1.TIF]
